# The Magnitude of Wasting and Associated Factors among Children Aged 2-5 Years in Southern Ethiopia: A Cross-Sectional Study

**DOI:** 10.1155/2021/6645996

**Published:** 2021-05-07

**Authors:** Selamawit Dires, Moges Mareg

**Affiliations:** Department of Reproductive Health, School of Public Health, College of Medical and Health Sciences, Dilla University, Dilla, Ethiopia

## Abstract

**Background:**

Malnutrition accounts for almost half of the global under-five child mortality. Worm infections are one of the immediate and commonest causes that affect the nutritional status of children. There is limited data related to the magnitude of wasting and associated factors among children. Therefore, this study was aimed at assessing the magnitude of wasting and associated factors among children aged 2 to 5 years in the Wonago district of Gedeo Zone, southern Ethiopia.

**Methods:**

Community-based cross-sectional study design was conducted. A total of 3324 children aged 2-5 years were included in the study. A pretested semistructured questionnaire was used for data collection, and anthropometric measurements were computed using the World Health Organization Anthro-nutritional software. The multivariate logistic regression analyses with adjusted odds ratio, 95% confidence interval, and *P* value less than 0.05 were used to identify the factors significantly associated with wasting.

**Results:**

A total of 3273 children aged 2-5 years participated with a 98.5% response rate. The magnitude of wasting was 13%. The factors like government-employed fathers [AOR = 1.93; 95% CI (1.08, 3.46)], child's age range between 48 and 59 months [AOR = 1.46; 95% CI (1.01, 2.09)], being a male child [AOR = 1.42; 95% CI (1.07, 1.88)], having diarrheal disease in the past two weeks [AOR = 0.39; 95% CI (0.17, 0.90)], and bathing less than two times per week [AOR = 1.42; 95% CI (1.03, 1.96)] were factors significantly associated with wasting.

**Conclusion:**

Undernutrition in children is still a problem, and the proportion of wasting was 13%. Government-employed fathers, child's age range between 48 and 59 months, being a male child, having diarrheal disease in the past two weeks, and bathing below two times per week were significantly associated with children's nutritional status warranting close attention by policymakers and stakeholders. For researchers, a further longitudinal study is recommended to get strong evidence.

## 1. Background

Nutritional status is the state of the body in a way that is influenced by the food that a person consumes [1]. The nutritional status of children is one of the most important indicators of a household's living standard and determinant of child survival, and it is a strong reflection of countries' development [2, 3].

The nutritional status of individuals and population groups is assessed by anthropometric measures (anthropometry is the measurement of the human body). Well-known anthropometric measurements are weight, height, and midupper arm circumference (MUAC) [4]. Certain measurements are expressed as indices, which include weight for age (WFA), height for age (HFA), weight for height (WFH), MUAC for age, and body mass index (BMI) for age. Each index is documented as a *z*-score that describes how far and in what direction an individual's anthropometric measurement diverges from the median [4].

There are several and varied causes of malnutrition which are interrelated with each other and classified as the front line. Most of these related factors are inadequate food intake and disease which are determined by several essential factors like household food security, maternal/child-caring practices, political factors, cultural conditions, access to health services, and access to a healthy environment [5, 6].

The magnitudes of wasting were high in the age range of 12 and 24 months when dietary inadequacy and diarrheal illnesses are most common [7]. It is connected with poor health in infants and young children. The consequence or complications of wasting are well-known. These are increased risk of illness from infectious diseases [8–10] and death [11, 12].

Research is needed on the risk factors of wasting at different ages and in different environmental and sociocultural settings. This is because wasting represents an acute form of undernutrition, and children who suffer from it face an increased risk of death, increased risk of diseases, and reduced productivity [13].

Globally, almost half (45%) of all deaths in under-five children were because of undernutrition. This problem puts children more to be prone to severe diseases and responsible for the unnecessary loss of three million young lives annually [14, 15]. A figure in 2014 showed that there were 50 million (8%) wasted children globally and from them, 16 million (2%) were severely wasted. On the other hand, about 151 million under-five children in 2017 suffer from chronic malnutrition and 67 million were wasted [16, 17].

In Africa in 2010, about 39.4%, 24.9%, and 10.3% of under-five children were stunted, underweight, and wasted, respectively [18]. In 2015, undernutrition in sub-Saharan Africa accounts for one-third of the global estimate [19]. The rate of malnutrition among children less than five years in Ethiopia is the highest from sub-Saharan and East African countries having 9% and 8.7% of wasted under-five aged children. It is also one of the most important causes of disease and mortality among under five children in the country [20, 21].

The trend from 2000 to 2014 in Ethiopia showed that there is a decrease in the prevalence of stunting by 31% and underweight by 39%. However, wasting declines only by a small proportion. Despite the results observed and ongoing attempts which have been done towards ensuring and minimizing under-five malnutrition, the nation is still facing a very high burden of undernutrition. According to 2014 Ethiopian minidemographic health survey (EMDHS) data, the national wasted children were 9% and severely wasted were 3% [22].

This national report also showed that the magnitudes of nutritional problems had regional variations, some with a higher/lower level than the national average including Southern Nations, Nationalities, and People's Region (SNNPR). Wasted children in this region, where the study area is found, were 7%, and severely wasted were 2% [22]. Moreover, there is a limited study on the magnitude and associated factors of wasting by age classification among under-five children in developing counties, especially in the study area. Therefore, the aim of this study was assessing the magnitude and factors associated with wasting in Wonago district, Gedeo Zone, southern Ethiopia.

## 2. Methods

### 2.1. Study Aim, Design, and Setting

A community-based cross-sectional study design was employed to assess the magnitude of wasting and associated factors among children aged 2-5 years with their mothers who were living in Wonago district. The district is found in the Gedeo Zone, Southern Nation Nationalities, and Peoples Region (SNNPR), Ethiopia. It is located 375 km southwest of Addis Ababa, the capital city of Ethiopia, 105 km south of Hawassa, and 12 km from Dilla, which is the provincial city of Gedeo Zone.

The district is one of the densely populated districts in Ethiopia. The 2015/2016 population data obtained from the district health office showed that the total population reached 145,635 and 29,720 households. Of the total population, 10.43% are children aged between 2 and 5 years old. There are 18 kebeles (the smallest administrative units of the Ethiopian governance system) in Wonago, one urban and 17 rural kebeles. There are six health centers, 20 health posts, two private clinics, three pharmacies, 33 elementary and junior schools, three high schools, and a preparatory school in the study area.

### 2.2. Sample Size and Sampling Procedure

*Sample size*: the study participants included a cohort of children aged 2-5 years of age who were living in the three selected clusters Hasse Haro (*n* = 983), Mokonissa (*n* = 1310), and Wonago kebeles (*n* = 1031).

(1) $$\begin{array}{c} N=983+1310+1031=3324. \end{array}$$

*Sampling procedure*: first, based on residential areas, the 18 kebeles in the woreda were taken as clusters, and from those, three kebeles were selected by lottery method, urban, and rural mix. The study participants were all children aged 2-5 years from these three clusters.

### 2.3. Data Collection Instruments and Procedure

Two types of instruments (questionnaire and anthropometric measure) were used to collect the required data. A pretested and validated semistructured interviewer-administered questionnaire was prepared in English and translated into Amharic and Gede-uffa and was used to obtain information on factors influencing the nutritional status of children. Besides, the anthropometric measure was used to estimate the nutritional status of the children.

Data were collected by those who can take anthropometric measures of a child and who spoke the local language (Gede-uffa). Data collectors were trained to measure height to the nearest 0.1 cm and weight with the subjects without shoes and in light clothes to the nearest 0.1 kg, using electronic weighing scales which were calibrated daily. To reduce measurement errors, the child's shoes, braids, and hair clips were removed before measurement. A stadiometer and digital weighing machine were used for height and weight measurement, respectively. In the process of anthropometric measurements, the child was in a stand-up position with feet together and flat on the ground, heels tetchy the back plate of the stadiometer, legs straight, buttocks against the backboard, scapula against the backboard, and arms loosely at their side.

### 2.4. Data Analysis

The collected data was entered in to computer using EpiData software, and analysis was done using Statistical Packages for Social Sciences (SPSS) version 20 software. The data was cleaned, and data integrity was checked. The standard score of WFH and proportions of wasting were estimated. The mother's BMI was determined and used as a factor that may affect the nutritional status of their children. A bivariable logistic regression was used to estimate the relationship of independent determinants of wasting. A multivariable logistic regression model was fitted to identify factors that are associated with wasting by adjusting for the possible confounders. Statistical precision was estimated with a 95% confidence interval, and statistical significance was declared at a P value ≤ 0.05.

The quality of data was ensured by using a digital weight scale, and by calibrating it daily, training of data collectors according to the standard of the WHO measurement techniques, close supervision, and prompt feedback were provided. The data were checked in the field to ensure that all the information was properly collected and recorded. Moreover, a sort of brief daily activity evaluation method was established to correct problems that could be faced during data collection. The consistency of data was maintained through the translation of the questionnaire into two local languages (Amharic and Gede-uffa) and back to English. Before and during data processing, the information was checked twice by two independent people for completeness.

### 2.5. Operational Definition

Weight-for-height *z* − score < −2 indicates wasting or thinness of children which reflects an acute weight loss, often associated with current starvation and/or severe disease, and it can also be the result of a chronic condition [22].

Undernutrition is a result of food scarcity and/or repeated infectious diseases. It includes stunting, wasting, and underweight [22].

Over nutrition is because of excessive food intake or excessive fat accumulation that presents a risk to health. It includes both overweight (weight-for-height *z* − score > +2 and ≤+3 *z*-score) and obesity (weight-for-height *z* − score > +3*z*-score) [22].

Well-nourished children are those whose *z*-score lies between >-2 and ≤+2 [22].

Sometimes sick refers to the sickness of mother on the pregnancy of the index child once but no hospitalization.

Sever/frequently sick stands for the sickness of mother on the pregnancy of the index child once and leads to hospitalization or more than one-time sick health experience.

### 2.6. Ethical Clearance

Ethical clearance (Ref. No. 009/2009) was obtained from Dilla University, College of Medicine and Health Sciences research review board (IRB), and a support letter from the school of Public Health was submitted to the Gedeo Zone Health Department and Wonago District health office, and permission was also obtained. The purpose of the study was explained to the mothers, and written consent was obtained from them. They were reassured about the safety of the procedure and maintaining confidentiality about the data they gave.

## 3. Results

A total of 3324 children who were aged 2-5 years during the deworming campaign from three kebeles, namely, Hasse Haro (*n* = 983), Mokonissa (*n* = 1310), and Wonago (*n* = 1031) were included. The mean age of mothers of index child (*n* = 3256) was 30 years (±SD = 5.72), many of them (95%) were married, 90% were Gedeo, 92.8% were protestant followers, and 88.9% were Gede-uffa speakers. The mean age of fathers of index child (*n* = 3114), who were the household heads, was 38 years (±SD = 8.71). Most of the study participants lived in rural kebeles (86%), in a nuclear type of family (88%), and a family size of 5-7 (55%). A large number of mothers (74%) and fathers (47%) had no formal education while large numbers of mothers (77%) and fathers (71%) were housewives and farmers, respectively. About 94% of households' heads were male in which 92% of household heads' relationship with the child is being a father. The average household monthly income was 555 Ethiopian birr, and their main source of food (76%) was mainly from their farming. The majority of the household heads (96%) own their plot of land whereas only 27% of them own livestock (Table 1).

About 57% of children in the study areas were born at home, while 41% were in a health institution and 0.5% was in other places. The mean birth order of the index children was 3 (±SD = 1.64). Most index children (92%) were born before the 6^th^ birth order. The mean birth interval of the index children was 2 years (±SD = 0.94). Slightly above half of the index children (53%) had ≤2 years of the birth interval from their preceding siblings. The majority of the index children (84%) had a sibling whose age is less than 5 years. Above two-thirds of index children (68%) got baby bath twice or more per week whereas 31% of the children have got once per week or not at all. The majority of children (81%) were previously dewormed, at least once. Moreover, almost all children (99%) ever took Bacillus Calmette-Guérin vaccine (BCG); polio 0, 1, 2; Penta 1, 2, 3, and measles vaccines. Besides, about a small number of children (6%) took another type of vaccine. Some of the children had an episode of illnesses in the previous two weeks just before baselines such as diarrhea (6%), acute respiratory infection (ARI) (5%), and fever (11%) (Table 1).

Above three-quarters of children (79%) feed three or more times per day. A large number of children breastfed for two or more years whereas there were no special prelacteal feeding practices in the study area. The mean period of exclusive breastfeeding was 5 months (±SD = 1.78). The children were exclusively breastfed either for less than six months (49%), for exactly six months (32%), or greater than six months (18%). After six months, children who got supplementary food by spoon-feeding were 45%. Less than half of the mothers of index children (42%) fed with extra food during pregnancy of the index child (Table 2).

The mean age of children was 40 months (±SD = 10.5); the mean weight was 12.64 kg (±SD = 2.37); and the mean height was 89.48 cm (±SD = 9.34). The majority (77%) of children were well-nourished, and about 13% of children were wasted.

### 3.1. Factors Associated with Wasting

Children residing in urban settings were 1.77 times more likely [AOR = 1.77 (95% CI: 1.35, 2.32)] to be wasted than rural residents. Children whose household heads' age is greater than 37 years were 1.25 times more likely [AOR = 1.25 (95% CI: 1.01, 1.55)] to be wasted than children with household heads' age equal to 37 and fewer years. Children who were among the extended family were 1.52 times more likely [AOR = 1.52 (95% CI: 1.13, 2.05)] to be wasted than those who lived in a nuclear family.

Merchant mother's children were 1.48 times more likely [AOR = 1.48 (95% CI: 1.05, 2.08)] to be wasted than housewife mothers. Children whose fathers are government-employed were 1.93 times more likely [AOR = 1.93 (95% CI: 1.08, 3.46)] to be wasted than those whose father's occupation was farmers. Family decisions made by other than family members like aunt and uncle were 2.56 times more likely [AOR = 2.56 (95% CI: 1.00, 6.52)] to be wasted than those made by the father. Children from households without land were 1.61 times more likely [AOR = 1.61 (95% CI: 1.01, 2.59)] to be wasted than children with households of their own. Children who live in households with livestock were 1.27 times more likely [AOR = 1.27 (95% CI: 1.00, 1.59)] to be wasted than those living in households that had no livestock.

Children whose age is between 36-47 months and 48-59 months were 1.36 [AOR = 1.36 (95% CI: 1.04, 1.78)] and 1.46 times more likely [AOR = 1.46 (95% CI: 1.01, 2.09)] to be wasted, respectively, than children whose age was between 24 and 35 months. Being a male child was 1.42 times more likely [AOR = 1.42 (95% CI: 1.07, 1.88)] to be wasted than females. Children whose birth interval was >2 years were 27% less likely to be [AOR = 0.73 (95% CI: 0.55, 0.98)] wasted than whose birth interval was ≤2 years (Table 3).

Children who did not take Penta_1_ and Penta_2_ vaccines were 3 times more likely [AOR = 3.01 (95% CI: 1.07, 8.48)] to be wasted than those who took the vaccine. Similarly, children who took other types such as the Rotavirus and pneumococcal congested vaccine were 1.62 times more likely [AOR = 1.62 (95% CI: 1.10, 2.36)] to be wasted than those who did not take other types. Children who had a diarrheal disease in the past two weeks from the study period were found to have 61% less likely [AOR = 0.39 (95% CI: 0.17, 0.90)] to be wasted when compared to those who did not have the disease. Children's feeding mechanism of additional food using a bottle were 1.8 times more likely [AOR = 1.80 (95% CI: 1.08, 3.01)] to be wasted than those whose way of feeding was using the hand.

Children whose mothers' BMI < 18.5 kg/m^2^ were 1.28 times more likely [AOR = 1.28 (95% CI: 1.03, 1.59)] to be wasted than children whose mothers' BMI ≥ 18.5 kg/m^2^. Children whose mothers attended at least 1 ANC follow-up were 33% less likely [AOR = 0.67 (95% CI: 0.46, 0.98)] to be wasted than those whose mothers' ANC follow-up was 4 and above. Children who had less than two times bathing per week were 1.4 times more likely [AOR = 1.42 (95% CI: 1.03, 1.96)] to be wasted than those with two and greater times bathing per week (Table 4).

## 4. Discussion

The study was aimed at assessing the magnitude and associated factors of wasting among children 2-5 years in Wonago district, southern Ethiopia. The study found that most, 77%, of the children were well-nourished and about 13% of children were wasted.

Despite the results observed and ongoing attempts which have been done towards ensuring and minimizing under-five malnutrition, Ethiopia is still facing undernutrition as a major health problem. The result of the current study also revealed that the percent of wasted children was above the national level reported by Ethiopian Demographic Health Survey (EMDHS) 2014, and EDHS 2016 data, respectively [20, 22]. This holds with regional levels too, which might be due to sample size variation.

The result of this study shows that children of government-employed fathers have an increased likelihood of being wasted than children of a farmer. This can be discussed as farm work makes fathers more familiar with food products and to be concerned about food consumption of children whereas government-employed fathers are far away from food concerns.

For the current study, the age group of children of 48-59 months was found to have an increased risk of wasting than 24-35-month age group children. This may be for the reason of the mobile nature of the children in the age group of 48-59 months and the need for high energy requirements. Being a male child has also increased the likelihood of wasting than females in this study which shows a consistent finding with the study in Haramaya district, eastern Ethiopia [23]. This is probably due to the fact that boys are involved in more energy-consuming tasks than girls.

Children who have diarrheal disease were found to be less likely to develop wasting than their counterparts. This result contradicts with the study on Bule Hora and Somalia which states that diarrheal disease experienced by children in the past two weeks before the data collection was an independent determinant factor for wasting to be developed [24, 25]. This may be due to the community trend variation in emphasizing for diseased children which helped them to reduce wasting.

In the current study, children with less bathing frequency per week were found to be more wasted as compared to those children with more frequent bathing. The reason for this might be due to inadequate sanitation that increases the probability of infectious diseases, which indirectly can cause malnutrition. This result may be argued since as the frequency of sanitation increases, the nutritional wellbeing of the child increases.

### 4.1. Strength and Limitation

This study collected data from all participants rather than sampling which increased the precision of estimates. The study collected information by questionnaire and anthropometric measures with daily calibration to minimize potential information bias. This study also has limitations. First, laboratory investigation and related data were not collected in this study. Second, other unmeasured confounders or residual confounders could play by inflating or underestimating the reported findings; multilevel modeling was not done. Finally, still, some measurement errors and recall biases could exist among respondents answering questions relating to events happening in the past.

## 5. Conclusion

The proportion of wasting was higher as compared to the national figure. Child, maternal, paternal, or environmental-related factors were significantly associated with child nutritional status. It was also found that government-employed fathers, child age group 48-59 months, being a male child, having the diarrheal disease in past two weeks before the data collection, and bathing frequency below two times per week were significant determinant variables of wasting for children with the 24-59-month age group. There is a need to focus on other causes like feeding less nourishing foods. Having a better sanitation habit is recommended. The age of the child has a significant effect on wasting. Hence, attention is needed on the education of child age-specific feeding and child care interventions.

## Figures and Tables

**Table 1:** Child's birth-related and health care characteristics in previous two weeks before the interview of the index child in Wonago district from December 19^th^ to 23^th^ (*n* = 3273).

Variable	Frequency *N* (%)
Place of birth of the child	3273 (100)
Home	1886 (57.6)
Health institution	1372 (41.9)
Other	15 (0.5)
Child's birth order group	3273 (100)
<6	3037 (92.8)
≥6	236 (7.2)
Mean (±SD)	2.94 (1.64)
Birth interval category preceding the child	2562 (100)
≤2 years	1364 (53.2)
>2 years	1198 (46.8)
Mean (±SD)	2.43 (0.94)
No. of U5 other than the child	1684 (100)
1 child	1420 (84.3)
>1 child	264 (15.7)
Mean (±SD)	0.6 (0.65)
Current deworming status of the child	3273 (100)
Yes	3273 (100)
No	0 (0.0)
Previous deworming frequency	3273 (100)
0	606 (18.5)
1	417 (12.7)
2	912 (27.9)
3	1306 (39.9)
4	32 (1.0)
Mean (±SD)	1.92 (1.14)
Any type of vaccine experience	3273 (100)
Yes	3273 (100)
No	0 (0.0)
BCG	3273 (100)
Yes	3256 (99.5)
No	17 (0.5)
Polio_0_	3273 (100)
Yes	3256 (99.5)
No	17 (0.5)
Polio_1_	3273 (100)
Yes	3256 (99.5)
No	17 (0.5)
Polio_2_	3273 (100)
Yes	3256 (99.5)
No	17 (0.5)
Penta_1_	3273 (100)
Yes	3255 (99.5)
No	18 (0.5)
Penta_2_	3273 (100)
Yes	3255 (99.5)
No	18 (0.5)
Penta_3_	3273 (100)
Yes	3255 (99.5)
No	17 (0.5)
Measles	3273 (100)
Yes	3244 (99.1)
No	29 (0.9)
Any other type of vaccine	3273 (100)
Yes	210 (6.4)
No	3063 (93.6)
Diarrhea experience	3273 (100)
Yes	197 (6.0)
No	3076 (94.0)
ARI experience	3273 (100)
Yes	188 (5.7)
No	3085 (94.3)
Fever experience	3273 (100)
Yes	362 (11.1)
No	2911 (88.9)

*N*: number; SD: standard deviation; BCG: Bacillus Calmette-Guérin vaccine; Penta: diphtheria, pertussis, tetanus vaccine; ARI: acute respiratory infection.

**Table 2:** Feeding practices of the index child and mothers during pregnancy of the index child, in Wonago district from December 19^th^ to23^th^ (*n* = 3273).

Variable	Frequency *N*^∗^ (%)
Frequency of daily meal	3273 (100)
≥3	2601 (79.5)
<3	672 (20.5)
Mean (±SD)	2.85 (0.57)
Years of GBF	3273 (100)
≥2 years	2876 (87.9)
<2 years	397 (12.1)
Mean (±SD)	2.1 (0.59)
Prelacteal feeding experience	3273 (100)
Yes	3273 (100)
No	0 (0.0)
No. of months for EBF	3273 (100)
<6 months	1610 (49.2)
6 months	1070 (32.7)
>6 months	593 (18.1)
Mean (±SD)	5.4 (1.78)
Mechanism of feeding	3273 (100)
Bottle	317 (9.7)
Cup	1245 (38.0)
Spoon	1482 (45.3)
Other	229 (7.0)
Use of extra food on pregnancy	3256 (100)
Yes	1388 (42.6)
No	1868 (57.4)

*N*: number; SD: standard deviation; GBF: general breastfeeding; EBF: exclusive breastfeeding.

**Table 3:** A bivariable and multivariable logistic regression model for determinants of wasting among children aged 2-5 years in Wonago district (*n* = 3273).

Variables		WHZ		COR (95% CI)	AOR (95% CI)
		Wasted	Not wasted		
Age of the mother	<30 years	160	1397	0.81 (0.65, 1.01)	0.87 (0.58, 1.31)
	≥30 years	209	1478		

Marital status of the mother	Married	353	2740		
	Single	1	16	0.49 (0.07, 3.67)	
	Widowed	6	60	0.78 (0.33, 1.81)	
	Divorced	9	59	1.18 (0.58, 2.41)	

Ethnic group of the mother	Gedeo	331	2607		
	Oromo	23	172	1.05 (0.67, 1.65)	
	Amhara	6	46	1.03 (0.44, 2.42)	
	Others	9	50	1.42 (0.69, 2.91)	

Religion of the mother	Protestant	332	2677		
	Orthodox	36	182	1.59 (1.09, 2.32)	0.99 (0.51, 1.91)
	Others	1	16	0.50 (0.07, 3.81)	0.36 (0.04, 3.15)

Language of the mother	Gede-uffa	320	2564		
	Amharic	44	199	1.77 (1.25, 2.51)	1.16 (0.57, 2.36)
	Others	5	112	0.36 (0.15, 0.88)	0.29 (0.09, 0.96)

Age group of father	≤37 years	166	1425		
	>37 years	188	1323	1.22 (0.98, 1.52)	0.19 (0.01, 4.26)

HHH's age group	≤37 years	177	1530		
	>37 years	196	1358	1.25 (1.01, 1.55)	6.52 (0.29, 146.70)

Residence of HH	Rural	295	2512		
	Urban	78	376	1.77 (1.35, 2.32)	1.91 (0.94, 3.89)

Type of family	Nuclear	312	2559		
	Extended	61	329	1.52 (1.13, 2.05)	1.16 (0.72, 1.86)

Family size category	2-4	109	923	0.50 (0.23, 1.11)	1.18 (0.39, 3.57)
	5-7	214	1594	0.57 (0.26, 1.25)	1.17 (0.43, 3.17)
	8-10	42	337	0.53 (0.23, 1.22)	0.88 (0.32, 2.44)
	>10	8	34		

Educational level of the mother	No formal education	269	2144		
	Primary	87	623	1.11 (0.86, 1.44)	
	Secondary	11	99	0.89 (0.47, 1.67)	
	Above secondary	2	9	1.77 (0.38, 8.24)	

Mothers' occupation	Housewife	269	2246		
	Farmer	46	319	1.20 (0.86, 1.68)	1.31 (0.81, 2.11)
	Merchant	44	249	1.48 (1.05, 2.08)	1.31 (0.79, 2.15)
	Government employee	1	25	0.33 (0.05, 2.48)	0.54 (0.06, 4.57)
	Others	9	36	2.09 (0.99, 4.38)	2.64 (0.89, 7.81)

The educational level of the father	No formal education	161	1303		
	Primary	135	975	1.12 (0.88, 1.43)	
	Secondary	45	394	0.92 (0.65, 1.31)	
	Above secondary	13	76	1.38 (0.75, 2.55)	

Fathers occupation	Farmer	241	1989		
	Merchant	58	463	1.03 (0.76, 1.40)	0.97 (0.64, 1.46)
	Government employee	26	116	1.85 (1.18, 2.89)	1.93 (1.08, 3.46)
	Others	29	180	1.33 (0.88, 2.01)	0.70 (0.38, 1.30)

HHH's sex	Male	353	2741		
	Female	20	147	1.06 (0.65, 1.71)	

HHH's r/p with the child	Father	336	2682		
	Mother	19	127	1.19 (0.73, 1.96)	1.22 (0.24, 6.17)
	Caregiver	13	59	1.76 (0.96, 3.24)	1.02 (0.41, 2.56)
	Other	5	20	1.99 (0.74, 5.35)	1.56 (0.13, 19.35)

HH's monthly income	≤500 birr	212	1555		
	>500 birr	161	1333	0.89 (0.71, 1.10)	

Family decision maker	Father	210	1612		
	Mother	40	241	1.27 (0.89, 1.83)	1.79 (0.91, 3.56)
	Both	117	1017	0.88 (0.69, 1.12)	1.23 (0.89, 1.69)
	Other	6	18	2.56 (1.00, 6.52)	1.29 (0.13, 12.68)

HH's source of food	Agricultural products	274	2201		
	Purchase	98	681	1.16 (0.90, 1.48)	0.76 (0.47, 1.23)
	Others	1	6	1.34 (0.16, 11.16)	

HH's land ownership	Yes	351	2780		
	No	22	108	1.61 (1.01, 2.59)	1.14 (0.53, 2.45)

HH's livestock ownership	Yes	118	773	1.27 (1.00, 1.59)	1.21 (0.87, 1.69)
	No	255	2115		

Age group of the child before	24-35 months	109	1079		
	36-47 months	128	931	1.36 (1.04, 1.78)	1.09 (0.76, 1.57)
	48-59 months	136	878	1.53 (1.17, 2.00)	1.46 (1.01, 2.09)

Sex of the child	Female	173	1605		
	Male	200	1283	1.45 (1.17, 1.79)	1.42 (1.07, 1.88)

Place of birth of the child	Home	208	1683		
	Health institution	165	1205	1.11 (0.89, 1.38)	

Child's birth order group	<6	348	2678		
	≥6	25	210	0.92 (0.59, 1.41)	

Birth interval category preceding the child	≤2 years	184	1175		
	>2 years	120	1075	0.71 (0.56, 0.91)	0.73 (0.55, 0.98)

No. of U5 other than the child	1 child	158	1255		
	>1 child	30	232	1.03 (0.68, 1.56)	

WHZ: weight-for-height *z*-score; COR: crude odds ratio; AOR: adjusted odds ratio; CI: confidence interval; HH: household; HHH: household head; r/p: relationship; No: number.

**Table 4:** A multivariable logistic regression model for determinants of wasting among children aged 2-5 years in Wonago district (*n* = 3273).

Variables		WHZ		COR (95% CI)	AOR (95% CI)
		Wasted	Not wasted		
Previous deworming frequency	0	34	572	0.58 (0.17, 1.98)	
	1	49	366	1.29 (0.38, 4.41)	
	2	120	788	1.47 (0.44, 4.91)	
	3	167	1133	1.43 (0.43, 4.73)	
	4	3	29		

BCG	Yes	369	2874		
	No	4	13	2.23 (0.73, 6.79)	0.79 (0.12, 5.14)

Polio_0_	Yes	370	2875		
	No	4	13	1.79 (0.51, 6.32)	

Polio_1_	Yes	370	2874		
	No	3	14	1.66 (0.48, 5.82)	

Polio_2_	Yes	370	2874		
	No	3	14	1.66 (0.48, 5.82)	

Penta_1_	Yes	368	2875		
	No	5	13	3.01 (1.07, 8.48)	4.54 (0.09, 214.49)

Penta_2_	Yes	368	2875		
	No	5	13	3.01 (1.07, 8.48)	

Penta_3_	Yes	369	2875		
	No	4	13	2.39 (0.78, 7.39)	0.74 (0.01, 42.33)

Measles	Yes	367	2865		
	No	6	33	2.04 (0.82, 5.03)	2.11 (0.56, 7.97)

Any other type of vaccine	Yes	35	174	1.62 (1.10, 2.36)	1.03 (0.54, 1.98)
	No	338	2714		

Diarrhea experience	Yes	17	180	0.72 (0.43, 1.19)	0.39 (0.17, 0.90)
	No	356	2708		

ARI experience	Yes	20	168	0.92 (0.57, 1.48)	
	No	353	2720		

Fever experience	Yes	41	321	0.99 (0.70, 1.39)	
	No	332	2567		

Frequency of daily meal	≥ 3	310	2279		
	< 3	63	609	0.76 (0.57, 1.01)	0.96 (0.64, 1.43)

No. of years for GBF	≥2 years	318	2546		
	<2 years	55	342	1.29 (0.95, 1.75)	1.01 (0.67, 1.51)

No. of months for EBF	<6 months	189	1414		
	6 months	115	951	0.91 (0.71, 1.16)	
	>6 months	69	523	0.99 (0.74, 1.32)	

Mechanism of feeding	Bottle	55	261	1.80 (1.08, 3.01)	1.40 (0.65, 3.02)
	Cup	142	1096	1.11 (0.70, 1.75)	1.36 (0.69, 2.64)
	Spoon	152	1326	0.98 (0.62, 1.54)	1.26 (0.65, 2.43)
	Hand	24	205		

Use of extra food on pregnancy	Yes	141	1244	0.81 (0.65, 1.01)	1.06 (0.75, 1.50)
	No	228	1631		

BMI	≥18.5 kg/m^2^	213	1830		
	<18.5 kg/m^2^	156	1045	1.28 (1.03, 1.59)	1.23 (0.92, 1.65)

Health status	Frequently sick	7	57	0.99 (0.45, 2.19)	0.76 (0.28, 2.07)
	Sometimes sick	78	523	1.21 (0.92, 1.58)	1.41 (0.98, 2.02)
	No sickness	284	2295		

No. of ANC visit	0	76	589	0.89 (0.64, 1.24)	0.89 (0.57, 1.38)
	1	47	480	0.67 (0.46, 0.98)	0.69 (0.42, 1.15)
	2	109	767	0.98 (0.72, 1.33)	0.94 (0.62, 1.44)
	3	55	475	0.79 (0.55, 1.15)	0.79 (0.49, 1.29)
	≥4	82	564		

FP methods information	Yes	322	2558		
	No	47	317	1.18 (0.85, 1.63)	

If yes, have you ever use it	Yes	286	2212		
	No	36	346	0.97 (0.75, 1.26)	

No. of bathing the child/week	**≥2**	242	2000		
	**<2**	131	888	1.22 (0.97, 1.53)	1.42 (1.03, 1.96)

Mothers handwashing after using toilet	Yes	318	2423		
	No	51	452	0.86 (0.63, 1.18)	

Mothers handwashing before meal preparation or serving	Yes	361	2811		
	No	8	64	0.97 (0.46, 2.05)	

HH's drinking water source	Well	173	1320		
	Pipe	166	1204	1.05 (0.84, 1.32)	0.82 (0.55, 1.20)
	Surface water	34	364	0.71 (0.49, 1.05)	0.76 (0.43, 1.34)

HH's window availability	Yes	298	2240		
	No	75	648	0.87 (0.67, 1.14)	

HH's condition of floor	Earth	328	2626		
	Dung	2	10	1.60 (0.35, 7.34)	0.00 (0.00)
	Cement	15	90	1.33 (0.76, 2.33)	0.98 (0.44, 2.17)
	Bamboo	28	162	1.38 (0.91, 2.10)	1.07 (0.54, 2.14)

HH's toilet availability	Yes	313	2393		
	No	60	495	0.93 (0.63, 1.24)	

If yes, what type of toilet is it	Open-pit	269	2064		
	Bush/field	19	198	0.74 (0.45, 1.19)	0.66 (0.37, 1.19)
	Pit with slab	19	105	1.39 (0.84, 2.30)	0.97 (0.49, 1.89)
	VIP	6	26	1.77 (0.72, 4.34)	1.35 (0.43, 4.26)

HH's garbage disposal pit availability	Yes	128	849	1.26 (0.99, 1.58)	1.03 (0.72, 1.47)
	No	245	2039		

HH's distance from the nearest health care service in km	>8.77 km	171	1300	1.03 (0.83, 1.28)	
	≤8.77 km	202	1588		

WHZ: weight-for-height *z*-score; COR: crude odds ratio; AOR: adjusted odds ratio; CI: confidence interval; BCG: Bacillus Calmette-Guérin vaccine; Penta: diphtheria, pertussis, tetanus, haemophilus influenza type B, hepatitis B vaccine; ARI: acute respiratory infection; No: number; GBF: general breastfeeding; EBF: exclusive breastfeeding; BMI: body mass index; ANC: antenatal care; FP: family planning; HH: household; VIP: ventilated pit latrine; km: kilometer.

## Data Availability

Data will be available upon request from the corresponding author.
